# Downregulation of respiratory complex I mediates major signalling changes triggered by TOR activation

**DOI:** 10.1038/s41598-020-61244-3

**Published:** 2020-03-10

**Authors:** Raquel Perez-Gomez, Valentina Magnin, Zorana Mihajlovic, Vera Slaninova, Alena Krejci

**Affiliations:** 1 Czech Academy of Sciences, Biology Centre, Institute of Entomology, Ceske Budejovice, Czech Republic; 2 University of South Bohemia, Faculty of Science, Ceske Budejovice, Czech Republic

**Keywords:** Cell signalling, Drosophila

## Abstract

Mitochondrial dysfunctions belong amongst the most common metabolic diseases but the signalling networks that lead to the manifestation of a disease phenotype are often not well understood. We identified the subunits of respiratory complex I, III and IV as mediators of major signalling changes during *Drosophila* wing disc development. Their downregulation in larval wing disc leads to robust stimulation of TOR activity, which in turn orchestrates a complex downstream signalling network. Specifically, after downregulation of the complex I subunit *ND-49* (mammalian *NDUFS2*), TOR activates JNK to induce cell death and ROS production essential for the stimulation of compensatory apoptosis-induced proliferation within the tissue. Additionally, TOR upregulates Notch and JAK/STAT signalling and it directs glycolytic switch of the target tissue. Our results highlight the central role of TOR signalling in mediating the complex response to mitochondrial respiratory dysfunction and they provide a rationale why the disease symptoms associated with respiratory dysfunctions are often alleviated by mTOR inhibitors.

## Introduction

Mitochondria play an essential function in cellular energetic and NADH metabolism. Five protein complexes (complex I-V) of the electron transport chain (ETC) have distinct functions in the oxidation of NADH and/or FADH_2_, maintenance of the inner mitochondrial membrane potential and production of ATP via oxidative phosphorylation. Moreover, they serve as signalling hubs for specific cellular events including ROS-mediated signalling, apoptosis and Ca^2+^ signalling^1^. Mutations in mitochondrial enzymes are the most frequent metabolic mutations present in human^2^.

Complex I of the ETC is the node point in the mitochondrial NADH metabolism as it mediates electron transfer from NADH to the other respiratory complexes^3^. Therefore, complex I inhibitors have been exploited as therapeutic targets in cancer treatment^4,5^, although the mechanism of action is often unclear. On the other hand, complex I inhibition can lead to increased proliferation, depending on the cell type and complex I inhibitor used^6–8^. Despite the fact that mitochondrial electron transport chain disorders are one of the most common human genetic diseases^9^, the mechanisms behind the dichotomy in the functional outputs after complex I inhibition are not well understood.

The mTOR pathway (TOR in *Drosophila*) is the key integrator of cellular metabolic inputs that connects cell growth with environmental signals, including nutrient and growth factors availability^10^. It promotes cell growth by stimulation of cellular translation, anabolic metabolism and by inhibiting autophagy. At the same time, mTOR activation can lead to apoptosis in certain contexts^11–13^. The upregulation of mTOR is observed during epithelial wound healing^14^, during aging as well as in many types of cancers^15^. Although strong inhibition of mitochondrial respiration can cause a metabolic catastrophe and cell death connected with mTOR inhibition through the activation of AMPK^16,17^, increasing evidence suggest that many types of respiratory dysfunctions are connected with increase in mTOR activity and mTOR inhibition leads to alleviation of the phenotype^18,19^.

We found that downregulation of mitochondrial respiratory complex I, III or IV stimulates TOR activity that directs major downstream signalling events, including Notch activation, metabolic changes and apoptosis-driven proliferation. As TOR overactivation balances between stimulation of apoptosis and proliferation, the model we present suggests a possible mechanism for the observations when complex I inhibition promotes either cell death or proliferation in different contexts. The signalling network we identified also suggests a possible explanation why the disease symptoms associated with respiratory dysfunctions are often alleviated by mTOR inhibitors.

## Results

### Downregulation of respiratory complexes stimulates TOR activity and its downstream signalling events

**Figure 1:**
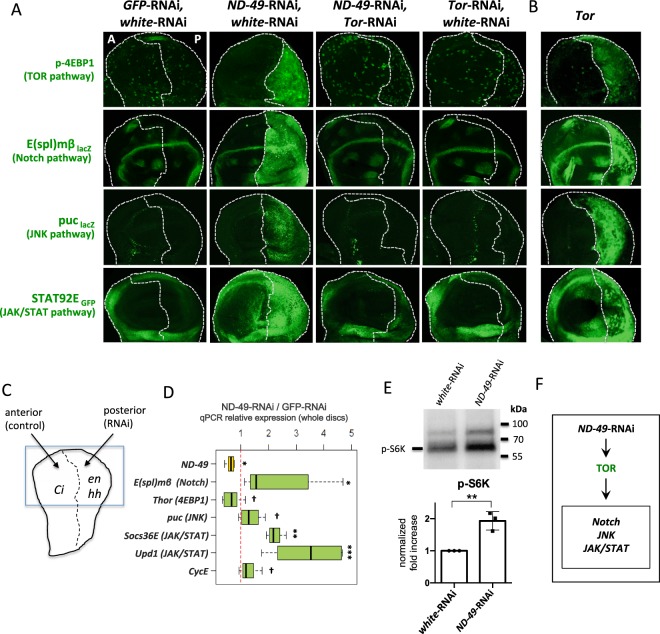
Increase in TOR activity orchestrates signalling changes after complex I downregulation. (**A**) Signalling changes caused by downregulation of respiratory complex I (RNAi against *ND-49* subunit) are rescued by downregulation of Tor pathway (Tor-RNAi). RNAi was driven only in the posterior part of the wing disc (*hh*-Gal4, Gal80^ts^ or *en*-Gal4 drivers), corresponding to the right halves of the immunostaining pictures. White dotted line defines the boundary between the anterior A and posterior P domains. The anteriror (left) half of the pictures serves as control. (**B**) The similarities between signalling changes caused by downregulation of respiratory complex I (*ND-49*-RNAi) and after overactivation of TOR in the posterior compartment (right half) of the wing disc. (**C**) The schematic overview of the immunostaining experiments. RNAi or other UAS experimental construct is driven in the posterior domain of the wing disc, the anterior domain serves as control. Blue frame depicts the wing pouch region shown in the figure panels. The boundary between the anterior and posterior domains was always determined by immunostaining of the Ci protein. (**D**) mRNA expression in the wing discs measured by qPCR. The RNA was extracted from the whole wing discs but only the posterior compartments were treated with RNAi, therefore the actual changes in gene expression in the posterior compartment are bigger than values plotted in the graph [***p < 0.001; **p < 0.01, *p < 0.05; ^†^p < 0.1]. (**E**) Western blot with antibody against p-S6K, another TOR target, detected in the *en-Gal4, ND-49-RNAi* and control (*en-Gal4, white-RNAi*) wing discs. Total protein load was assessed by fluorescent detection of protein lanes within the TGX Stain-Free gel (Biorad, Figs. S1D and S6). **p < 0.01. (**F**) Graphical summary of Fig. 1.

To characterize the signalling changes during respiratory dysfunction we downregulated complex 1 subunit, ND-49 (mammalian orthologue of NDUFS2) by RNAi in the Drosophila wing disc. We drove the RNAi only in the posterior domain of the disc by the *hh*-Gal4 driver so as we could use the anterior domain as a control (Fig. 1C). We observed upregulation of several signalling pathways, including Tor, Notch, JNK and JAK/STAT pathways, based on an increase in their reporter activities (Fig. 1A), as well as mRNA upregulation of their targets or ligands (Fig. 1D). In contrast, activity of the EGFR pathway remained unchanged (Fig. S1B). Thus, activity of some, but not all signalling pathways are upregulated by complex I depletion. Similar changes in signalling pathways were observed after downregulation of another subunits of complex I, as well as downregulation of respiratory complex III or IV, suggesting that the phenotype we observe is attributable to overall general respiratory dysfunction (Fig. S1A).

Surprisingly, the TOR pathway was upregulated after downregulation of complex I subunits (Fig. 1A,E) or subunits of complex III and IV (Fig. S1A). Tor activation is associated with increased levels of the phosphorylated, inactive, form of the negative translational regulator 4EBP1 protein (p-4EBP1) that is the classical target of Tor signalling. The increase in p-4EBP1 can at the same time be accompanied by transcriptional downregulation of 4EBP1 mRNA expression^20,21^. As apparent from Figs. 1A and S1A, we observed a strong increase in the levels of p-4EBP1 in the posterior region of wing discs, the area in which the subunits of respiratory complex I, III or IV had been downregulated (see also Fig. S1C for second RNAi line verification). Accordingly, the mRNA for *4EBP1* (*Thor* in *Drosophila*) was also downregulated after *ND-49*-RNAi treatment (Fig. 1D). As expected, the increase in p-4EBP1 was fully rescued by inhibition of the *Drosophila* TOR pathway via *Tor*-RNAi (Fig. 1A). To further verify TOR pathway overactivation after *ND-49-RNAi* we detected higher levels of p-S6K, another target of TOR pathway, on a western blot from *ND-49-RNAi* wing disc (Fig. 1E).

The key question is how the downregulation of respiratory complexes I, III or IV brings about the profound signalling changes and how they relate to each other. Strikingly, we obtained identical signalling and growth phenotypes by overactivation of the TOR pathway in the wing disc (Fig. 1B). Moreover, all of the *ND-49-*RNAi phenotypes were fully rescued by *Tor*-RNAi (Fig. 1A). These results strongly suggest that TOR signalling stimulated by dysfunctional respiratory chain is the initial element triggering all of the other signalling changes (Fig. 1F).

### Downregulation of complex I mediates compensatory apoptosis-induced proliferation that is dependent on JNK and effector caspases

**Figure 2:**
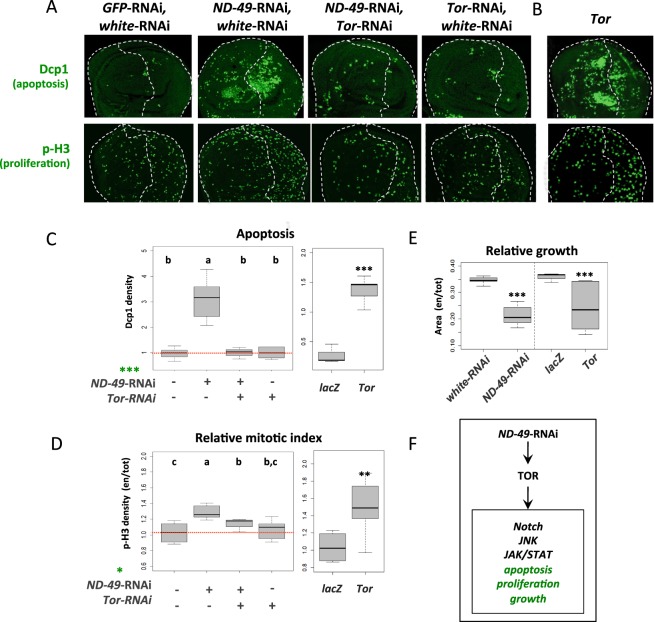
The effect of complex I downregulation on apoptosis, proliferation and tissue growth is mediated by Tor. (**A**) Induction of apoptosis (Dcp1) and proliferation (p-H3) after *ND-49*-RNAi in the posterior domain (right half) of the wing disc is rescued by *TOR*-RNAi. (**B**) Overexpression of Tor in the posterior domain leads to the same phenotype as *ND-49*-RNAi. (**C**) Quantifications of apoptosis (Dcp1), (**D**) the relative mitotic index (p-H3) and (**E**) the size of posterior wing disc compartment after *ND-49*-RNAi, *Tor*-RNAi and *Tor* overexpression. We detect interaction with the effect of *ND-49* knockdown using ANOVA [***p < 0.001; *p < 0.05]. *LacZ* represents control. (**F**) Graphical summary of Fig. 2.

We next monitored effects on cell death and proliferation during respiratory dysfunction. After downregulation of complex I, apoptosis (Dcp1) was strongly triggered in distinct areas of the wing pouch, both cell autonomously and non-autonomously (Figs. 2A,C and S1A). There was also a significant increase in proliferation as evident from the elevated levels of phosphorylated histone H3 (p-H3, Figs. 2A,D and S1A) and Cyclin E gene expression (Fig. 1D). The specific dCP1 and p-H3 patterns in individual discs were variable, probably reflecting the dynamic apoptotic and proliferation events, but they clearly showed increases of the signals in *ND-49*-RNAi wing discs. Similar increase in apoptosis and proliferation occurred after downregulation of complex III or IV (Fig. S1A) and also after Tor overexpression (Fig. 2B–D), supporting our view that TOR triggers the downstream signalling changes during respiratory dysfunction. As expected from this respect, the increase in apoptosis and proliferation after *ND-49*-RNAi was rescued by *Tor*-RNAi (Fig. 2C,D). The net result from the simultaneous increase in apoptosis and proliferation was that the posterior domains of the wing disc were significantly smaller than in controls (Fig. 2E).

**Figure 3:**
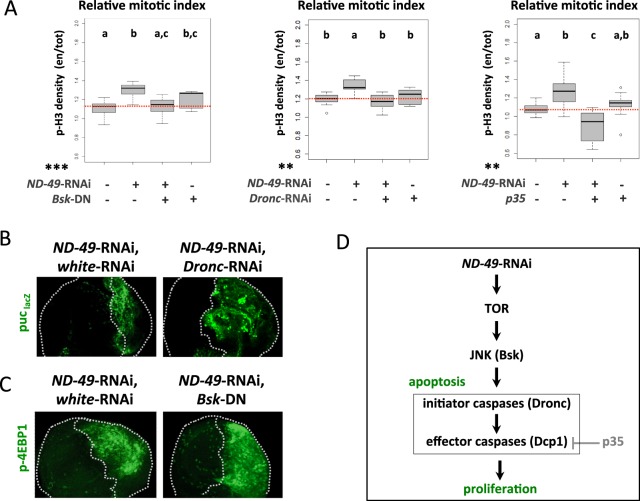
Downregulation of complex I mediates JNK driven compensatory apoptosis-induced proliferation, dependent on effector caspases. (**A**) Proliferation following *ND-49*-RNAi in the posterior compartment of the wing disc (right half) can be rescued by blocking the JNK pathway (through dominant negative *Bsk*^*DN*^), the initiator caspase (*Dronc*-RNAi) or the effector caspases (by overexpression of p35); we detect interaction with the effect of *ND-49* knockdown in cell proliferation using ANOVA [***p < 0.001; **p < 0.01; *p < 0.05]; a Duncan test was performed and groups that are statistically different were assigned using letters (p < 0.05; a, b, c). A genotype is assigned to two groups when it is not significantly different from any of them. (**B**) Blocking the initiator caspase by *Dronc*-RNAi in the posterior compartment in the context of *ND-49*-RNAi does not rescue the activity of the JNK pathway reporter *puc*-lacZ. (**C**) Blocking the JNK pathway by overexpression of *Bsk*^*DN*^ in the posterior compartment (right half) of the wing disc does not reduce the increased activity of TOR pathway (p-4EBP1) in the context of *ND-49*-RNAi. (**D**) Graphical summary of Fig. 3.

As downregulation of respiratory complexes I, III and IV in the wing disc caused simultaneously cell death as well as proliferation within the same tissue (Figs. 2 and S1A) we decided to test whether these two events are functionally connected via the mechanism of apoptosis-induced proliferation (AIP)^22,23^. Under such scenario the proliferation would be mediated via a non-apoptotic role of either the initiator^24,25^ or effector caspases^26^, which would signal from the dying cells to stimulate compensatory proliferation of their neighbours. Indeed, when we blocked apoptosis at the level of the initiator caspase Dronc by RNAi (or by overexpression of Diap1, Fig. S2A), or when we blocked the effector caspases by overexpression of p35 protein^27^, we rescued the increase in proliferation caused by downregulation of *ND-49* (Fig. 3A). These results indicate that proliferation following downregulation of complex I is dependent on apoptosis and it relies on the activity of effector caspases.

**Figure 4:**
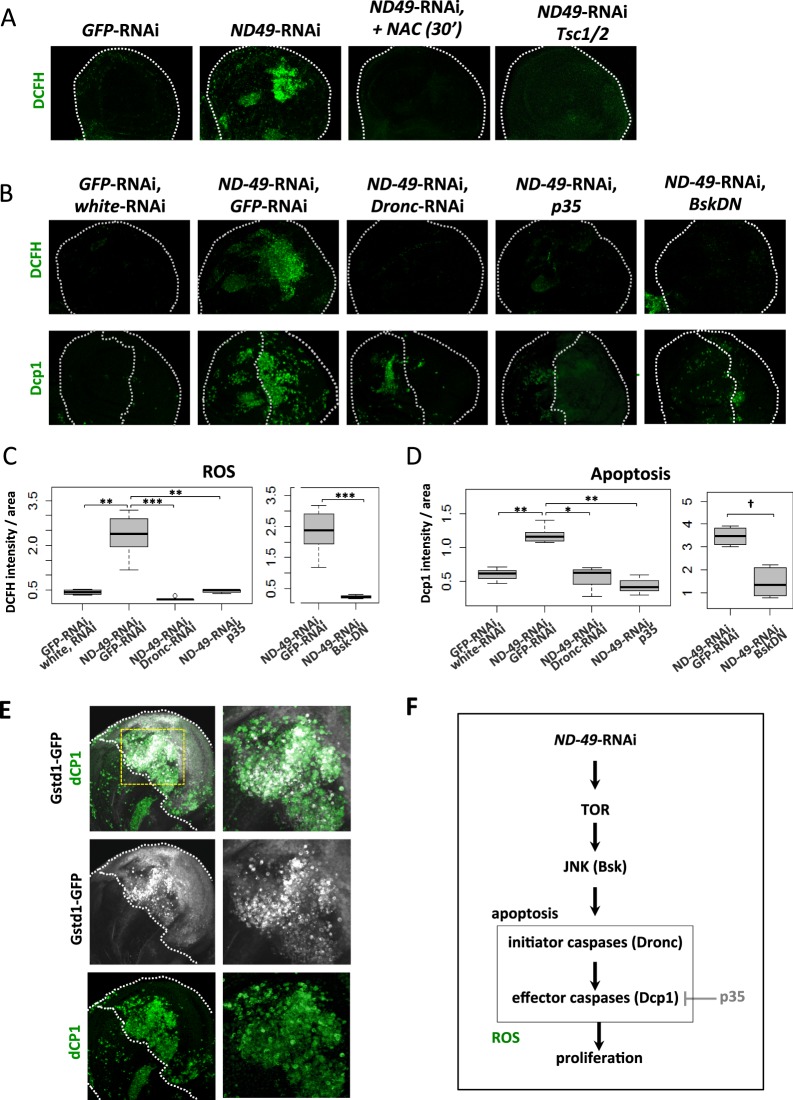
Strong formation of ROS after complex I downregulation is connected with apoptosis and is mediated by JNK. (**A**) Downregulation of *ND-49* in the posterior compartment of the wing disc causes ROS production (DCFH) that is rescued by incubation with the ROS scavenger N-acetylcysteine (NAC). Posterior domain is located on the right halves of the pictures (exact position of the A/P boundary could not be determined in this experiment). (**B**) Downregulation of *ND-49* in the posterior compartment of the wing disc causes ROS production (DCFH) as well as apoptosis (Dcp1) that are rescued by simultaneous block of the initiator caspase (*Dronc*-RNAi), by blocking the effector caspase (overexpression of p35*)* or the JNK pathway (dominant negative Bsk). (**C,D**) Quantification of data presented in panel (B). (**E**) The ROS sensitive reporter GstD1-GFP (grey) shows low level of activity within the whole posterior domain and a burst of signal in the same region of the disc where apoposis is occuring (dCP1, green). Yellow box indicates magnified area of the disc to see cellular resolution of the signal. (**F**) Graphical summary of Fig. 4.

In agreement with TOR pathway being the main functional effector triggered by complex I downregulation, we were able to rescue the tissue proliferation by *Tor-*RNAi (Fig. 2D) or by expressing the TOR negative regulators Tsc1 and Tsc2 (Fig. S2A). Similarly to a previously described model of AIP in *Drosophila* wing disc, the proliferation after *ND-49*-RNAi was dependent on the activity of JNK, as we were able to rescue the increased proliferation by overexpression of dominant negative *Bsk* (Fig. 3A). However, the JNK activity in our model must be functionally upstream of the apoptotic events because blocking the initiator caspase by *Dronc*-RNAi did not eliminate JNK activity (Fig. 3B) and because Bsk on its own was able to induce apoptosis (Fig. S2B). Moreover, as overexpression of dominant negative *Bsk* or *Bsk*-RNAi did not affect TOR activity (Figs. 3C and S2C) and Bsk alone could not activate TOR (Fig. S2B), we can place JNK in our model downstream of TOR activation but upstream of apoptosis (Fig. 3D). However, as apoptosis and ROS production are two inseparable events after *ND-49*-RNAi (as we show later in Fig. 4), we cannot exclude that the proliferation is dependent not only on the function of effector caspases but also on the production of ROS.

As TOR is upstream of AIP in our model we were curious to know if other well established models of AIP^24,28,29^ also involve TOR signalling. When we overexpressed UAS-*Hid* in the posterior domain of the wing disc (to also elicit AIP, as previously reported), we induced cell death and ROS formation but we did not observe the increase in p-4EBP1 staining (Fig. S2E), suggesting that TOR pathway activation is unique to our model of AIP.

Finally, we asked what was the relation between cell death and the activity of other signalling pathways we had recorded after down-regulation of *ND-49*. As apparent from Fig. S2D the TOR, Notch, JNK and JAK/STAT signalling were unchanged after we blocked the effector caspases by p35 in our model, suggesting that all these signalling events are upstream or independent of apoptosis.

### JNK triggers apoptosis and ROS formation after downregulation of respiratory complex I

Complex I, along with complex III, are considered the main sites of mitochondrial ROS production under conditions when electron flow is perturbed^30^. However, it is known that the type of complex I inhibition dictates whether ROS will be produced or not^31^. Using three different ROS sensitive dyes and the ROS sensitive *GstD1*-GFP reporter, we reproducibly observed increased ROS production in specific areas of the wing disc after downregulation of *ND-49* subunit (Figs. 4A,B, and S3A–C). Moreover, the ROS signal was not detected after incubation of discs with the ROS scavenger N-acetylcysteine (NAC) and it was dependent on TOR activation (Fig. 4A). The ROS localized to the same region of the wing pouch as apoptosis (Fig. 4B,E). Importantly, downregulation of the initiator caspase Dronc or the effector caspases (by overexpression of p35 protein) completely blocked the ROS burst associated with downregulation of *ND-49* (Fig. 4C). Moreover, only a subset of dCP1 positive cells showed a strong GstD1-GFP signal (Fig. 4E). Collectively, this data support the idea that ROS burst might be downstream of activation of caspases, as described in other models of AIP^32,33^, but additional experiments are needed to answer this interesting question. The stability of the GstD1-GFP reporter allowed us to visualise that a weak ROS signal is present in all cells of the posterior domain after *ND-49*-RNAi, probably as a consequence of complex I disruption (Fig. 4E). Nevertheless, the results indicate that the sites of strong ROS production we detect in our model are not produced by the primary complex I dysfunction but rather that they accompany apoptosis.

We further asked what is responsible for mediating the apoptotic events and ROS production consequent to downregulation of *ND-49*. As the JNK pathway is upregulated in these discs and it was described in numerous other contexts to trigger apoptosis^34^ we tested whether overexpression of Bsk, the *Drosophila* ortholog of JNK, was sufficient to trigger apoptosis and ROS production in the wing disc. Indeed, both were elevated during Bsk overexpression, as predicted by this model (Fig. S2B). Importantly, blocking the JNK pathway using dominant negative Bsk (Fig. 4B–D) or by expressing *Bsk*-RNAi (Fig. S3C) rescued both the apoptosis and ROS generation from the *ND-49*-RNAi mediated knockdown. Regions of strong production of ROS following complex I knockdown therefore appear to be consequences of apoptosis driven by JNK activation.

### Downregulation of complex I leads to stimulation of glucose metabolism and to changes in mitochondrial morphology

**Figure 5:**
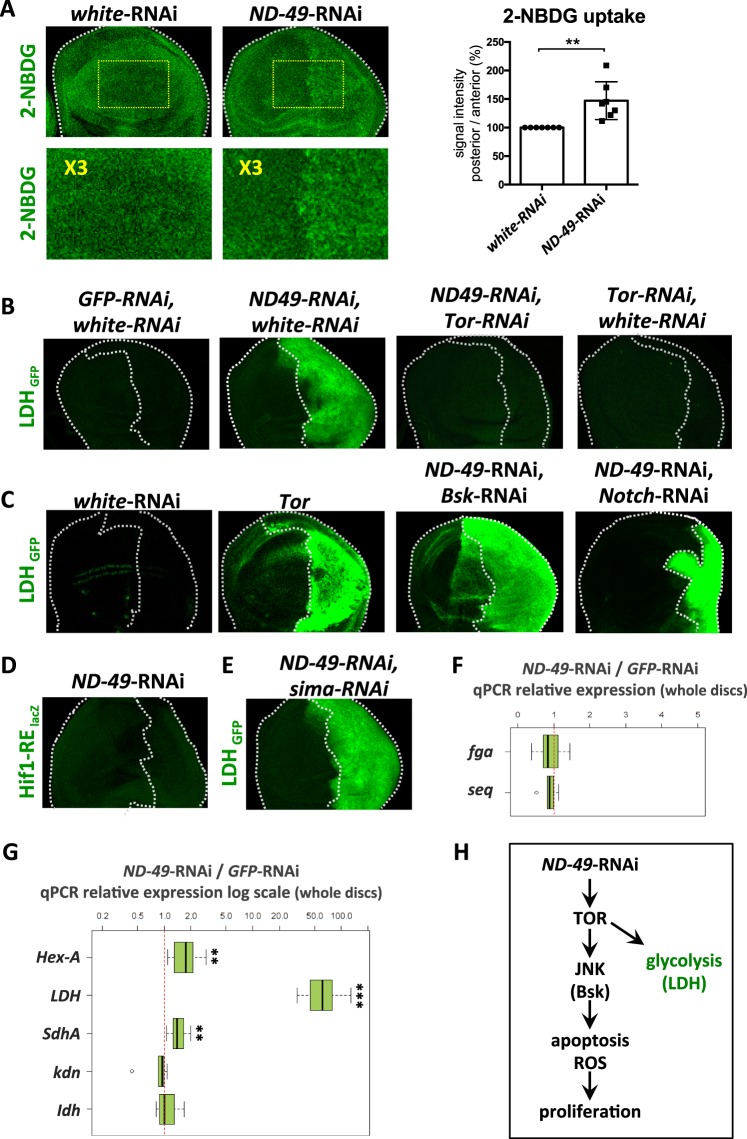
Downregulation of complex I leads to stimulation of glucose metabolism downstream of TOR. (**A**) The uptake of fluorescently labelled 2-deoxyglucose (2-NBDG) is increased after *ND-49* knockdown in the posterior part of the wing disc. Lower panel shows magnification of the region marked by yellow frame. **p < 0.01 (**B**) *Ldh* expression is increased after *ND-49* downregulation in the posterior part of the wing disc and this increase is rescued by *Tor-RNAi*. (**C**) Ldh expression is induced following Tor overexpression but it is not rescued by *Bsk*-RNAi or *Notch*-RNAi. (**D**) Hif-1 responsive element reporter (Hif1-RE-lacZ) is not active after *ND-49*-RNAi in the posterior part of the wing disc. (**E**) RNAi against *sima*, the *Drosophila* homologue of mammalian HIF-1, can not rescue Ldh increase after *ND-49*-RNAi in the posterior domain. (**F**) Transcription of Hif-1 transcriptional targets (*fga*, *seq*) is not changed after *ND-49-*RNAi in the posterior domain. (**G**) Downregulation of *ND-49* in the posterior compartment stimulates transcription of *Hexokinase A* (*HexA*) and lactate dehydrogenase (*Ldh*), as well as the *SdhA* subunit of respiratory complex II. Transcription of the TCA cycle genes (*kdn, Idh*) is not changed. The RNA was extracted from the whole wing disc but only the posterior compartment was affected by RNAi, therefore the actual changes in gene expression in the posterior compartment are bigger than the values plotted in the graph [***p < 0.001; **p < 0.01]. (**H**) Graphical summary of Fig. 5.

To ensure the biosynthesis of building blocks needed for cell growth, proliferating tissues often undergo metabolic switch towards increased levels of glycolysis, connected with elevated uptake of glucose^35,36^. However, an accompanying enhancement of mitochondrial metabolism is not observed because the excess of pyruvate derived from upregulated glycolysis is diverted away from mitochondria by its conversion to lactate through the activity of lactate dehydrogenase^37^. After downregulation of *ND-49* we observed increased uptake of fluorescently labelled glucose (2-NBDG, Fig. 5A), as well as the transcriptional upregulation of hexokinase A (Hex-A), the key regulatory enzyme controlling entry of glucose substrate into glycolysis (Fig. 5G). Moreover, the expression of lactate dehydrogenase (Ldh) was also strongly stimulated by *ND-49*-RNAi (Fig. 5B,G); a result that could be phenocopied by the overactivation of Tor (Fig. 5C) and rescued by *Tor*-RNAi (Fig. 5B). On the other hand, expression of TCA genes remained unchanged (*kdn, Idh*, Fig. 5G). Taken together, these data confirm that downregulation of complex I in the wing disc tissue elicits mRNA expression changes that may contribute towards a glycolytic shift, characterized by elevated glucose consumption and high expression of lactate dehydrogenase that diverts pyruvate away from the mitochondrial metabolism.

In several other contexts, LDH induction and glycolytic shift is triggered by transcription factor Hif-1^38,39^. However, Hif1 activity is not induced in our model, based on the lack of the activity of the Hif1-RE reporter^40^ (Fig. 5D) and no induction of Hif1 targets genes *fga* and *seq* (Fig. 5F). Moreover, Ldh expression can not be rescued by *sima*-RNAi, the *Drosophila* homologue of HIF-1 (Fig. 5E). At the same time, Ldh induction is not abolished by inhibition of Notch or JNK pathways (Fig. 5C). As direct activation of TOR pathway induced Ldh but Ldh expression could not be rescued by blocking JNK or Notch during *ND-49*-RNAi (Fig. 5C), we conclude that the observed glycolytic shift after complex I downregulation is caused by TOR overactivation, independently of JNK and Notch induction (Fig. 5H).

We also wondered if downregulation of *ND-49* and the associated metabolic changes affect mitochondrial morphology and function. First of all, we confirmed lower O_2_ consumption after *ND-49* downregulation in the larva (Fig. S4E), in agreement with NDUFS2 downregulation in mammals^41,42^. Downregulation of another subunit of complex I, *ND-75* (*CG2286*, *NDUFS1* in mammals) in *Drosophila* neurons has been reported to give rise to enlarged mitochondria with abnormalities in their cristae^43^, consistent with studies in human and mouse tissues^44^. In agreement with these observations, we found that the mitochondrial mass was increased after *ND-49*-RNAi (Fig. S4A,B) but the amount of mitochondrial DNA remained unchanged (Fig. S4C). Although we can not exclude that dynamic mitochondria biogenesis/degradation is involved, our experiments are consistent with a situation where that mitochondria in *ND-49*-RNAi cells are enlarged but their number is kept constant. The mitochondrial membrane potential was not severely compromised after *ND-49*-RNAi based on MitoTracker Red or TMRE staining (Fig. S4D). It is well known that cells with compromised respiratory chain can sustain mitochondrial membrane potential through the reversal of the ATP synthase function (i.e. using ATP hydrolysis to actively pump protons across the inner mitochondrial membrane)^45^. In this case, treating cells with the ATP synthase inhibitor oligomycin should lead to decreased membrane potential^46^. In our case however, oligomycin treatment leads to an overall increase in membrane potential in the whole disc, suggesting that membrane potential is not maintained via the reverse electron flow (Fig. S4D). Instead, it could be maintained by the compensatory activity of complex II, as we detected an upregulation of mRNA for *SdhA* subunit of complex II in the wing discs (Fig. 5G).

### The Notch pathway is activated by TOR

**Figure 6:**
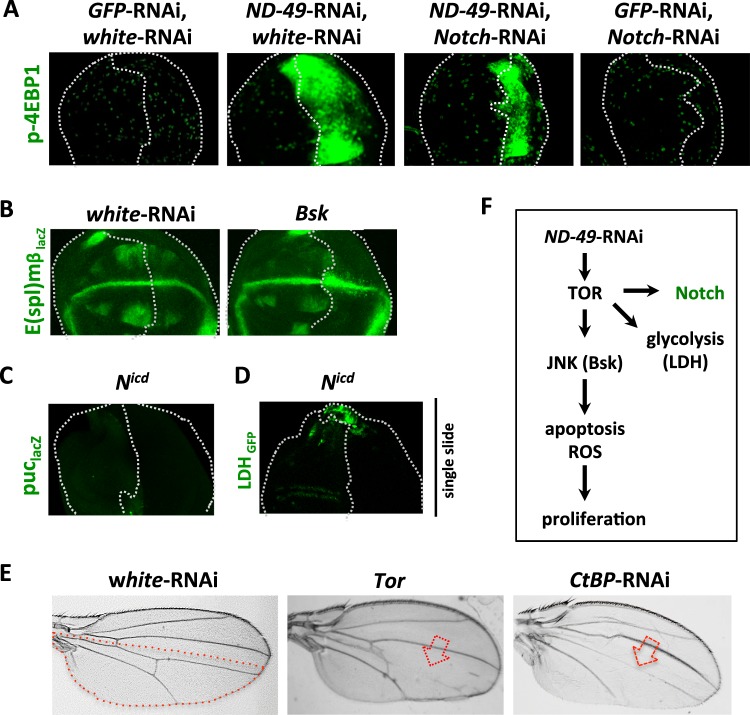
Notch pathway is activated by TOR after downregulation of complex I. (**A**) The induction of TOR pathway (p-4EBP1) after complex I downregulation in the posterior domain (right half) of the wing disc can not be rescued by *Notch*-RNAi. (**B**) Activation of JNK (*Bsk*) in the posterior domain has a negligible effect on the expression of the Notch target gene *E(spl)mβ* (compare to Fig. 1A). (**C**) Activation of Notch pathway by overexpression of Nicd in the posterior compartment does not stimulate JNK activity (*puc*-lacZ) nor Ldh expression (**D**). (**E**) TOR overactivation in the posterior compartment of the wing disc leads to loss of veins in adult wings, a typical Notch gain-of-function phenotype (compare to the RNAi against Notch repressor *CtBP*). Red dotted line represents the posterior compartment of the adult wing, red arrows point to the missing veins. (**F**) Overview of the signalling network triggered by *ND-49* downregulation in the wing disc.

Our original observations demonstrated that Notch signalling is enhanced after downregulation of respiratory complex I, III or IV (Figs. 1A and S1A), a phenotype that could be mimicked by Tor overexpression and rescued by *Tor*-RNAi (Fig. 1A). Notch activation alone was unable to induce the activity of JNK pathway (Fig. 6C) nor the expression of Ldh (Fig. 6D) to the extent of *ND-49* knockdown, and *Notch*-RNAi did not abolish activation of TOR (Fig. 6A). Overexpression of Bsk had a negligible effect on E(spl)mβ when compared to *ND-49*-RNAi alone (Figs. 1A and 6B). We infer that TOR is responsible for the Notch pathway activation following complex I downregulation, in parallel to activation of JNK and induction of glycolysis (Fig. 6F). In agreement, overactivation of the Notch pathway was unable to activate TOR, JAK/STAT or JNK signalling (Fig. S5A). However, overactivated Notch was sufficient to induce apoptosis and it may contribute therefore to the apoptosis-induced proliferation in our model, alongside JNK (Fig. S5A). Overactivation of TOR in the posterior domain of the wing disc leads to a loss of veins in adult wings, a typical Notch gain of function phenotype^47^, confirming the positive effect of TOR overactivation on Notch signalling (Fig. 6E).

Together these data confirm that Notch activity is upregulated via TOR following complex I downregulation and that other changes in signalling, such as JNK and stimulation of LDH expression, occur in parallel.

## Discussion

Despite the fact that mutations in mitochondrial enzymes are the most frequent metabolic mutations present in human, manifested in a whole range of clinical disorders^48^, the actual signalling networks that are triggered by malfunctioning mitochondria to develop clinical symptoms are still not well understood. Our results argue that TOR pathway is the key signalling effector triggered after downregulation of complex I, III or IV in the *Drosophila* wing disc. TOR activity in turn activates JNK, Notch and JAK/STAT signalling, boosts glycolysis and promotes compensatory apoptosis-induced proliferation to produce profound effects on tissue size and patterning.

By placing TOR at the top of the signalling network triggered by complex I dysfunction, we can provide a rationale for numerous observations where TOR inhibition alleviated the disease symptoms associated with mitochondrial dysfunction. For example, the maternally inherited Leigh syndrome (MILS), caused by mutation in complex I subunit Ndufs4, is associated with enhanced mTOR activity in neurons and the disease symptoms can be alleviated using chemical mTOR inhibitors^18,49^. Upregulation of mTORC1 has also been described as a key component of the mitochondrial integrated stress response during mitochondrial myopathy^19^. Alongside this line, the low survival rate of flies with mutation in the ND2 subunit of complex I can also be rescued by chemical TOR inhibition in *Drosophila*^50^. Moreover, an aggressive phenotype of breast cancer that is associated with complex I mutations can be reversed via restoration of complex I function that is associated with decreased mTOR activity^51^. Therefore, our data showing the role of TOR at the very apex of the signalling hierarchy after complex I dysfunction makes it interesting to test if similar regulatory mechanism underpins other types of mitochondrial dysfunction.

Respiratory inhibitors are used to supress various types of cancers^4,5^ although respiratory dysfunction can also promote cancer progression^6–8,44,52,53^. Based on our data we could hypothesize that the types of complex I dysfunctions that stimulate cancer progression would correlate with overstimulation of mTOR activity that initiates downstream signalling events promoting apoptosis but also apoptosis-induced proliferation. This may seem contradictory as complex I inhibition usually leads to decrease in ATP:ADP ratio that in turn activates AMPK, a known suppressor of mTOR activity^17,54^. However, the evidence that complex I inhibition would actually lead to downregulation of mTOR activity is surprisingly scarce and concerns mainly complex I inhibitors like biguanides (metformin, fenformin^5,16,55^) or fenofibrate^17^. On the contrary, there is ample of observations where mTOR is increased during mitochondrial dysfunction, supporting our results^18,19,49–51^. In fact, mTOR activity can be stimulated even in the presence of active AMPK, as in the case of the Leigh syndrome caused by mutation in complex I subunit Ndufs4^49^. By balancing between stimulation of apoptosis and proliferation, the mTOR driven signalling network we identified may suggest a possible mechanism for the contradictory observations where complex I inhibition was reported to promote cell death but also support proliferation depending on context.

One remaining question is how TOR is upregulated by mitochondria. One possibility may be an activation of TOR via mitochondrial Akt signalling and TOR complexes located in mitochondria-associated endoplasmic reticulum^56–58^. However, since TOR operates at the top of the signalling hierarchy after complex I downregulation, we can also speculate that its activity could be sensitive to the primary metabolic misbalance caused by disruption of mitochondrial metabolism. Indeed, the decrease in the NAD:NADH ratio and the associated slowdown of the TCA cycle that are associated with downregulation of respiration^59^ are likely to influence the activity of protein metabolic sensors such as sirtuin deacetylases or 2-KG dependent demethylases that may in turn regulate mTOR activity, either directly or indirectly, as suggested in some other contexts^60–64^.

Through non-apoptotic roles of caspases, dying cells can release diffusible mitogens and thus signal to their neighbours and instruct them to proliferate; a process known as apoptosis-induced proliferation (AIP)^25,32,65^. Several modes of AIP have been described in various species and tissues characterised by the use of either initiator or effector caspase to drive the signalling mechanism that in turn promotes proliferation. In *Drosophila* wing or eye discs, the most studied AIP models are based on targeted expression of the pro-apoptotic genes *hid* or *rpr* where the proliferation is dependent on the initiator caspases that activate ROS production and JNK activity^24,25,28,29,66,67^. In our model, proliferation is also dependent on caspases, ROS production and JNK activation, however it is unique in the way it is triggered and in the way the signalling components are interconnected: (1) it is initiated by downregulation of mitochondrial respiratory complex I and therefore it has a metabolic origin (2) it is orchestrated by consequent activation of the TOR pathway (3) it is dependent on effector caspases and (4) JNK activation is upstream of cell death, not activated by the non-apoptotic roles of caspases. Examples of AIP dependent on effector caspases have been described in *Drosophila* postmitotic cells in the eye disc^26^ and in mammalian cells after irradiation induced cell damage^68^ but the signalling components involved and their regulatory relationship also differ from our model.

It is important to stress that the high levels of ROS species are not produced in every cell where *ND-49*-RNAi is induced. Although low levels of ROS may appear as a primary consequence of *ND-49* downregulation, the strong ROS signal we observe in certain areas of the wing disc occurs downstream of cell death, as blocking apoptosis alongside *ND-49*-RNAi also eliminates the ROS signal. This is in agreement with ROS generation in other modes of AIP^22,32^. Although ROS production was described with certain complex I inhibitors^69^ it does not happen when other inhibitors are used^31^. Assembly of complex I into supercomplexes with other ETC proteins determines if ROS will be produced or not^70^. In our model it is obvious that the majority of ROS we observe does not originate from the dysfunctional complex I but they result from apoptosis, as blocking apoptosis prevents also ROS formation.

Taken together, our results highlight the central role of TOR pathway activation during mitochondrial dysfunction. As TOR overactivation gives identical phenotype to complex I downregulation, future studies should investigate if our results may be relevant outside of the mitochondria field, in some of the other contexts involving TOR overactivation, such as many types of cancer, wound healing or aging, with potentially important clinical implications.

## Materials and Methods

### *Drosophila* strains and genetics

All strains used in this work are described in Tables ST1, including reporters for signalling pathways. Expression in the posterior (right) half of the wing disc was driven by *hh-*Gal4, *Gal80*^ts^ (*hedgehog, III*.) or *en-*Gal4, *Gal80*^ts^ (engrailed, II.); anterior (left) part of the wing disc served as internal control. Crosses were kept at 29 °C, except a special regime for *UAS-Bsk* (25 °C and last 48 h at 29 °C), Tor (25 °C and last 60 h at 29 °C) and except *Tor*-RNAi, *Tsc1/2* and *Bsk*^*DN*^ crossed with *hh-Gal4,Tub-Gal80*^*TS*^ flies (25 °C and last 72 h at 29 °C). We used UAS*-white-*RNAi, UAS*-GFP-*RNAi or UAS-*lacZ* as stuffings to equalize the number of UAS constructs between control and experimental flies.

### Immunostaining and confocal imaging

Late third instar larvae were dissected, fixed in 4% formaldehyde for 40 minutes and stained according to a standard protocol. Primary and secondary antibodies used and their concentrations are listed in Table ST2. Staining against *Cubitus interruptus* (Ci) was always used as reference to define anterior and posterior domains (marked by dotted lines in the Figures, see also Fig. 1C). DAPI (SIGMA D9542-5MG, 1:5000) was added together with secondary antibodies. Visualization of JAK/STAT-GFP and NRE-RFP reporters do not require staining. Wing discs were mounted in Aqua Poly Mount media (Polysciences), pictures were taken at Olympus IX81 Confocal Laser Scanning Microscope and analyzed by Olympus FluoView FV1000 or ImageJ software. Identical laser and confocal settings were used throughout a single experiment that included control and experimental samples. All pictures represent stacks, except single slices in Figs. 6C and S4A.

### Measuring of ROS, mitochondrial membrane potential and glucose and O_2_ consumption

Wing discs from late L3 larvae were cultured in Shields and Shangs M3 insect medium (SIGMA, S3652) with dichloro-dihydro-fluorescein diacetate (DCFH, SIGMA 21884, 30uM), CellROX-DR (Thermofisher C10422, 1:500), Mitosox Red (Thermofisher, 10 µM), MitoTracker Red CMXRos (Thermofisher M7512, 25 nM), tetramethylrhodamine (TMRE, Thermofisher, 25 nM), N-acetylcystein (NAC, Sigma Aldrich A7250, 100ug/ml) or 2-(*N*-(7-Nitrobenz-2-oxa-1,3-diazol-4-yl)Amino)-2-Deoxyglucose (2-NBDG, Thermofisher N13195, 5 µg/ml) for 30 minutes at 25 °C before fix in 4% formaldehyde for 30 minutes, wash in PBS and mounting for confocal imaging.

To measure basal O_2_ consumption larvae from cross *Tub-*Gal4*, Tub-*Gal80^ts^ x *ND-49-*RNAi were kept at 29 °C for 72 h prior to dissection to induce *ND-49-*RNAi ubiquitously. Twenty L3 larvae were turned inside out and measured in respiration media (20 mM Tris-HCl, pH 7.5, 0.6 M Sorbitol, 2 mM EDTA, 0.03% Digitonin) using precalibrated Oroboros Oxygraph-2k respirometer. Values were normalized to larva weight and to control values from *GFP-RNAi* cross.

### Analysis of mRNA and mtDNA

Dissections of wing discs were performed in cold PBS within 15 minute interval, in 6 replicates per genotype using 50 wing discs per replicate. RNA was extracted by TRI reagent (Sigma Aldrich, T9424), treated with DNAse (Turbo DNA-free kit, Thermo Fisher Scientific, AM1906) and reverse transcribed with M-MLV reverse transcriptase (Sigma Aldrich, M170B). For quantifications of mitochondrial DNA content, 3 replicates of each genotype (50 discs each) were used for genomic DNA extraction.

Real-time PCR was performed using GoTaq qPCR master mix (Promega, A600A) on a Bio-Rad CFX96 machine. Primers were designed not to span introns and serially diluted genomic DNA from w^1118^ flies was used for calibration curve. mRNA expression was normalized to the mRNA levels of housekeeping gene *rp49*. The mtDNA content was analyzed after total genomic DNA extraction from the wing discs and plotted as the ratio between mitochondrial 16S rRNA/nuclear CG16941 relative to 16S rRNA/CG16941 in control *w*^1118^ flies.

### Western blot

For western blot analysis 20 wing discs were dissected in PBS on ice and homogenized in SDS sample buffer (0.2 M Tris pH 6.8, 50% glycerol, 10% SDS, 0.5% brophenol blue, 0.5% b-mercaptoethanol). The extract was heated at 100° for 3 min, fractionated on 4–20% Mini-PROTEAN TGX Stain-Free protein gel (Biorad) and blotted on PVDF Immobilon-P transfer membrane (Millipore). Total protein load was assessed by fluorescent detection of proteins within the TGX Stain-Free gel using the ChemiDoc imaging system (Biorad, Fig. S1D). The blot was probed with phospho-Drosophila p70-S6K antibody (Cell Signalling 9209, 1:1000) and detected with the Clarity western ECL kit (Biorad). Graph in Fig. 1E represent normalized values from three independent biological replicates. Uncropped original pictures included as Supplementary Fig. S6.

### Quantifications and statistics

Wing size quantification and bristle counting were performed with ImageJ (n ≥ 20) and genotypes were compared to their respective controls using Student’s t-test.

Quantifications of p-H3, Dcp1 and DCFH intensity were performed using ImageJ in 4–12 discs per genotype, we calculated the average intensity of signal per selected area. The p-H3 signal in posterior (*en*) domain was plotted relative to the signal from the whole disc; total mitotic rate varies strongly during wing disc development but relative one remains constant. Dcp1 quantifications are presented as signal density in posterior (*en*) domain; DCFH quantifications are plotted as signal density in the whole disc. Phenotypes in Figs. 1E, 2E, 4C,D, 5A, S3C and S4E were compared individually to their controls using t-test. Results in Figs. 2C,D, 3A and Fig. S2A were analyzed with ANOVA to detect interaction between genotypes. We also performed a Duncan test (p < 0.05) to compare the averages between treatments; the genotypes were assigned to different groups (a,b,c) when they were different from each other with 95% of confidence; one genotype can be assigned to two groups when it is not significanly different from any of them. We represent the mRNA quantification results as ratio of normalized *en-*Gal4>*ND-49-*RNAi expression relative to normalized *en-*Gal4*>GFP-*RNAi *expression* and analyze if they differ from 1 using Student’s t-test. Levels of mtDNA in *en-Gal4* > *ND-49-RNAi* and control *en-Gal4* > *white-RNAi* discs were compared with Student’s t-test.

All plots and statistics were performed using RStudio Version 0.98.1028. Ϯ p < 0.1; * p ≤ 0.05; ** p ≤ 0.01; *** p ≤ 0.001.

## Supplementary information

Supplementary figures and tables
