# Supplementary material for: Downregulation of respiratory complex I mediates major signalling changes triggered by TOR activation
Source: Sci Rep. 2020 Mar 10;10:4401. doi: 10.1038/s41598-020-61244-3 (PMC7064613; doi:10.1038/s41598-020-61244-3)
Supplement: Supplementary file 1 — Supplementary figures and tables [file 41598_2020_61244_MOESM1_ESM.pdf]

Downregulation of respiratory complex I mediates major signalling changes triggered by TOR activation

Raquel Perez-Gomez , Valentina Magnin , Zorana Mihajlovic , Vera Slaninova and Alena Krejci

SUPPLEMENTARY FILES S1 – S6

SUPPLEMENTARY TABLES ST1 AND ST2 AVAILABLE SEPARATELY

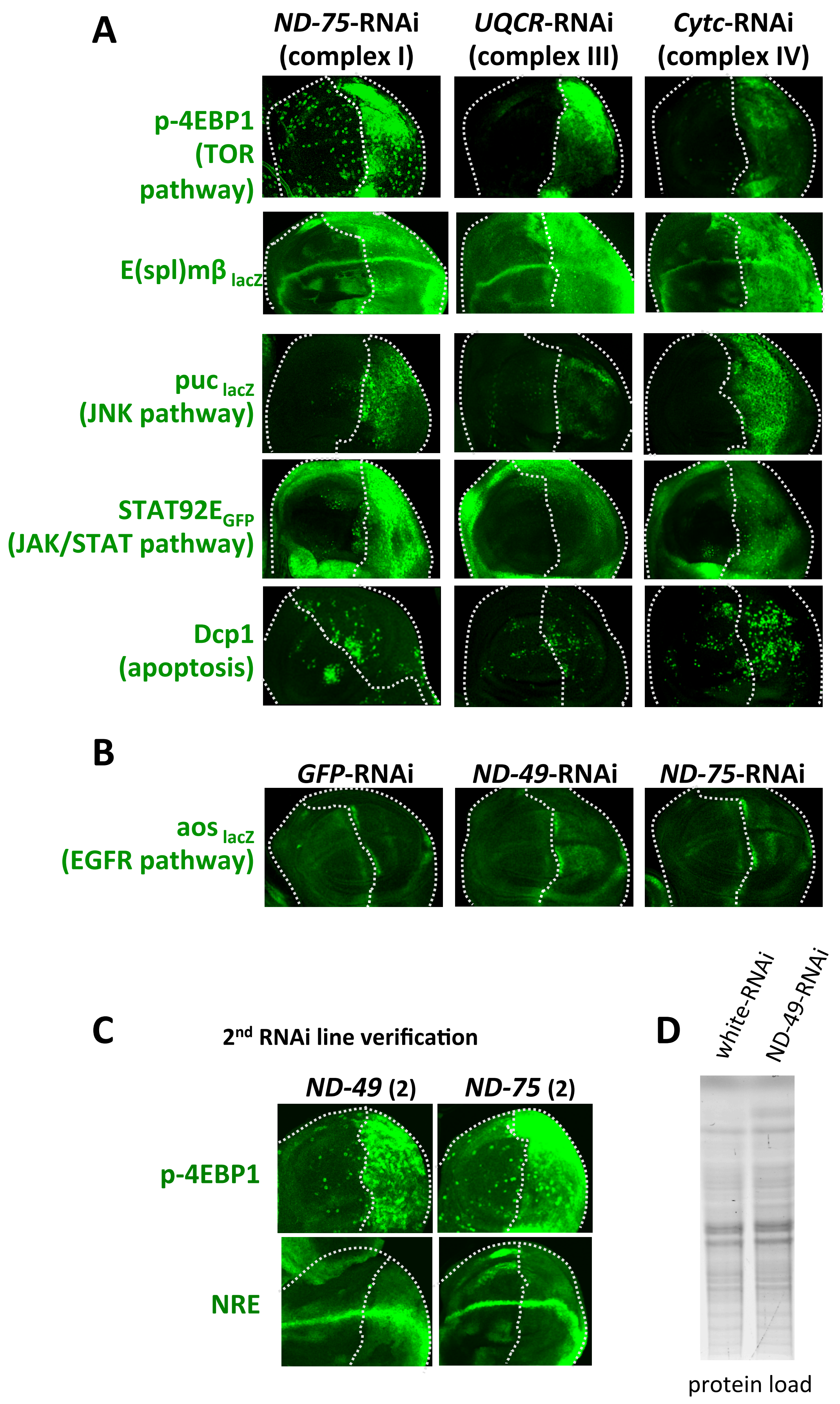

Supplementary figure S1: Downregulation of respiratory complex I, III or IV leads to similar downstream signalling changes.
(A) Downregulation of another subunit of respiratory complex I (*ND75*-RNAi) or the components of complex III and complex IV in the posterior (right half) compartment of the wing disc trigger similar signalling changes as downregulation of *ND-49* described in Fig.1. (B) The EGFR pathway is not affected after complex I downregulation. (C) Verification of the TOR and Notch pathway activation using different RNAi lines against *ND-49* or *ND-75* subunits of complex I. NRE stands for Notch responsive element reporter. (D) Total protein load for p-S6K wetstern blot from Fig. 1E, assessed by fluorescent detection of proteins within the TGX Stain-Free gel using the ChemiDoc imaging system (Biorad).

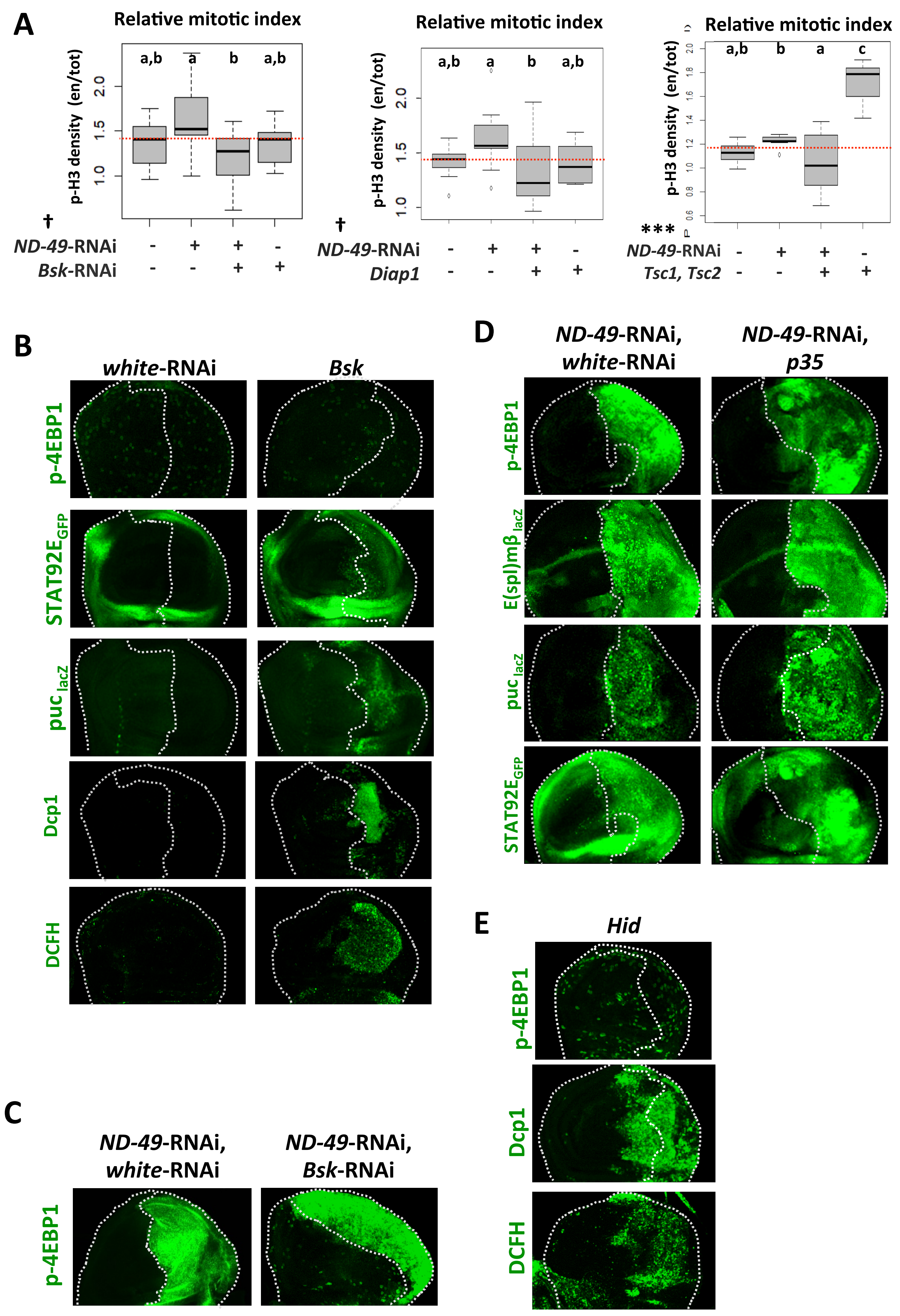
A
Relative mitotic index
p-H3 density (en/tot)
a,b
a
b
a,b
†
ND-49-RNAi
Bsk-RNAi
Diap1
c
***
Tsc1, Tsc2
B
white-RNAi
Bsk
p-4EBP1
STAT92E
puc lacZ
Dcp1
DCFH
C
ND-49-RNAi, white-RNAi
ND-49-RNAi, Bsk-RNAi
D
ND-49-RNAi, p35
E(spl)mβ lacZ
E
Hid

Supplementary figure S2: Apoptosis-induced proliferation is downstream of JNK and effector caspases in our model of complex I downregulation.

(A) Increased proliferation following the *ND-49*-RNAi in the posterior compartment of the wing disc is rescued by JNK inhibition (*Bsk*-RNAi), by inhibitor of caspases (expression of *Diap1*), or by TOR inhibition (expression of the negative Tor regulators Tsc1 and Tsc2). (B) The overexpression of *Bsk* in the posterior compartment does not activate Tor pathway (p-4EBP1) but it activates JAK/STAT (STAT92E-GFP), JNK (*puc*-lacZ) signalling, cell death (Dcp1) and ROS production (DCFH). (C) Blocking the JNK pathway by *Bsk*-RNAi does not eliminate TOR pathway activation (p-4EBP1) in the context of *ND-49*-RNAi. (D) Blocking the effector caspases by overexpression of *p35* does not affect TOR, Notch, JNK or JAK/STAT signalling elevated after the downregulation of complex I in the posterior compartment of the wing disc. (E) Expression of the proapoptotic gene *Hid* in the posterior compartment promotes apoptosis (Dcp1) and ROS production (DCFH) but not activation of Tor pathway (p-4EBP1).

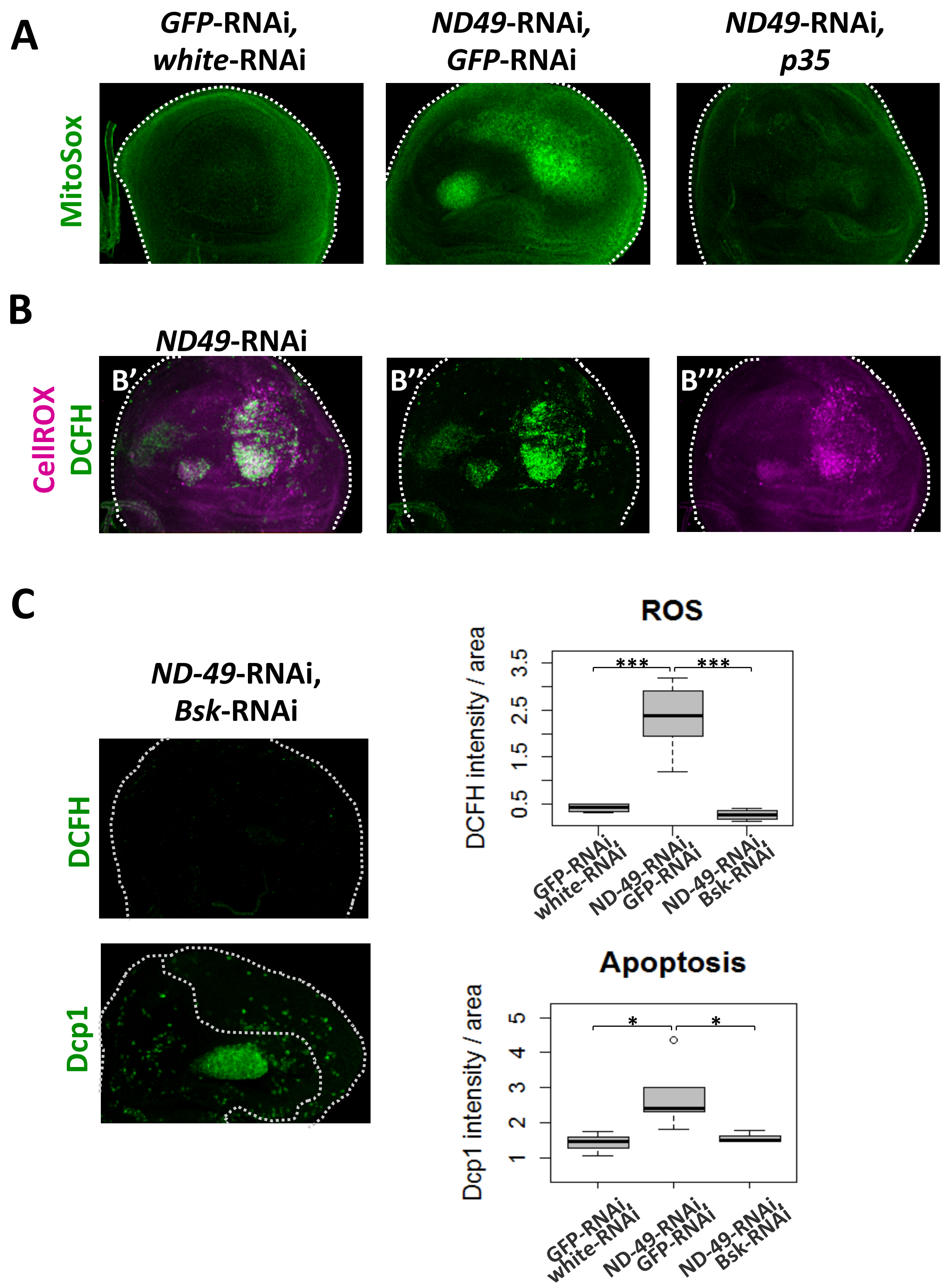

Supplementary figure S3: ROS production is associated with cell death in our model of complex I downregulation.

(A) Mitosox Red staining indicates ROS production in specific areas of the wing disc after downregulation of *ND-49* in the posterior domain that can be rescued by inhibiting the effector caspase by p35 expression. (B) Another ROS sensitive dye, CellROX (magenta), overlaps with DCFH staining (green). (C) Expression of *Bsk*-RNAi rescues the autonomous cell death in the posterior domain and ROS production after the downregulation of *ND-49* in the posterior compartment of the wing disc.

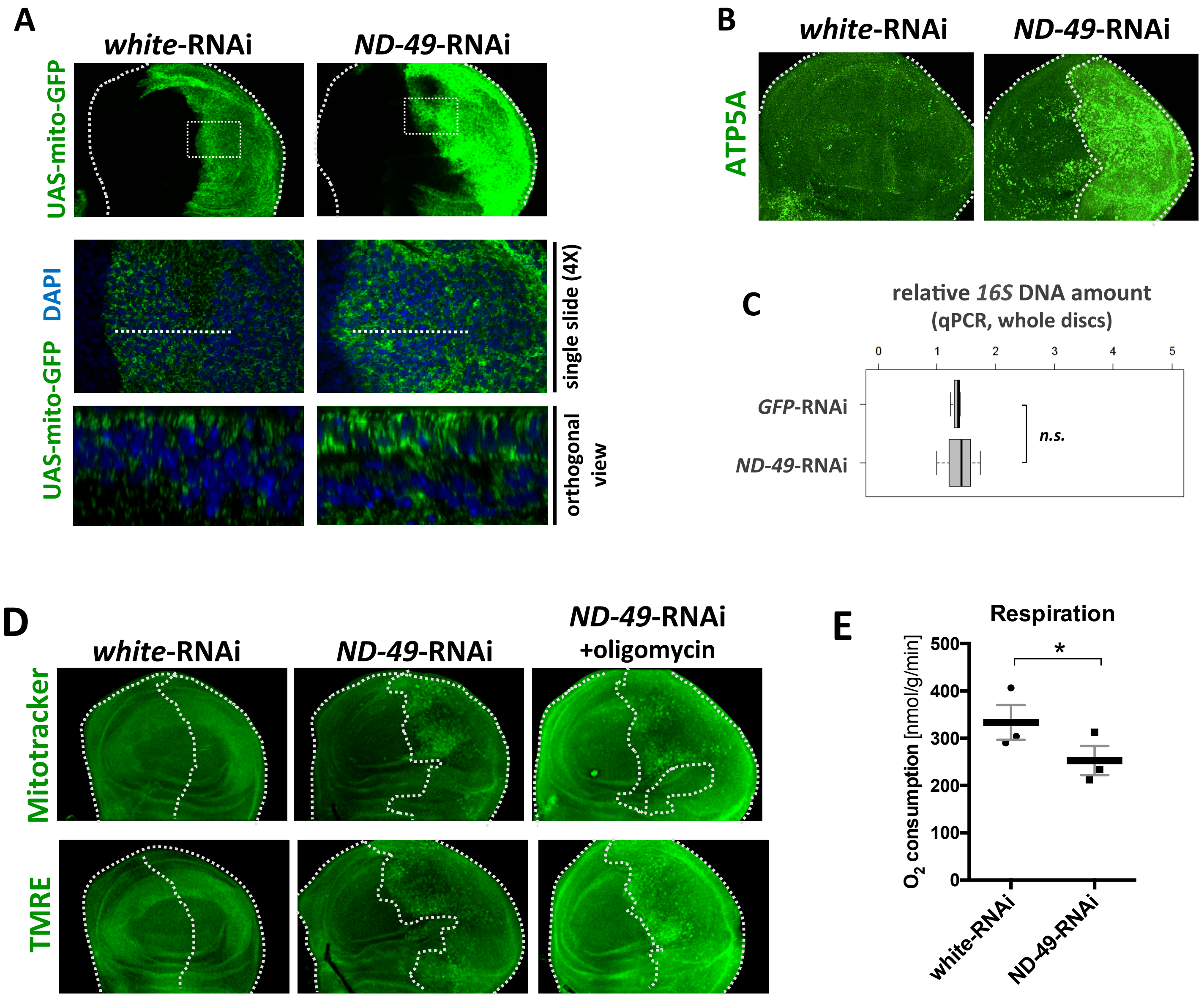

Supplementary figure S4: Changes in mitochondria and metabolism after downregulation of complex I. (A) The signal of the mitochondrial GFP (mito-GFP) reporter is elevated after downregulation of *ND-49* in the posterior compartment (right half) of the wing disc. Closer magnifications and orthogonal views reveal that mito-GFP signal fills most of the cytoplasm. (B) The amount of respiratory complex V (ATP5A) is elevated after downregulation of *ND-49* in the posterior compartment. (C) The relative amount of mtDNA (gene for *16S* rRNA) is not changed after *ND-49*-RNAi in the posterior part of the wing disc measured by qPCR. (D) Assessment of the mitochondrial membrane potential using MitoTracker Red and TMRE dyes. The signal in the posterior domain after *ND-49*-RNAi is not decreased in comparison to the anterior domain without RNAi induction, suggesting that membrane potential does not decrease after *ND-49* downregulation. The membrane potential in the whole disc is elevated after incubation of the wing disc with the ATP-synthase inhibitor oligomycin. The increase in control anterior domain is expected during this treatment. However, in the posterior domain with *ND-49* downregulation the membrane potential is also increased, suggesting that mitochondrial membrane potential is not maintained via reverse electron flow. (E) O_2 consumption after *ND-49*-RNAi in the whole larva, using the *Tub*-Gal4*, Tub*-Gal80^{ts} driver. Student's t-test, [* $p<0.05$].

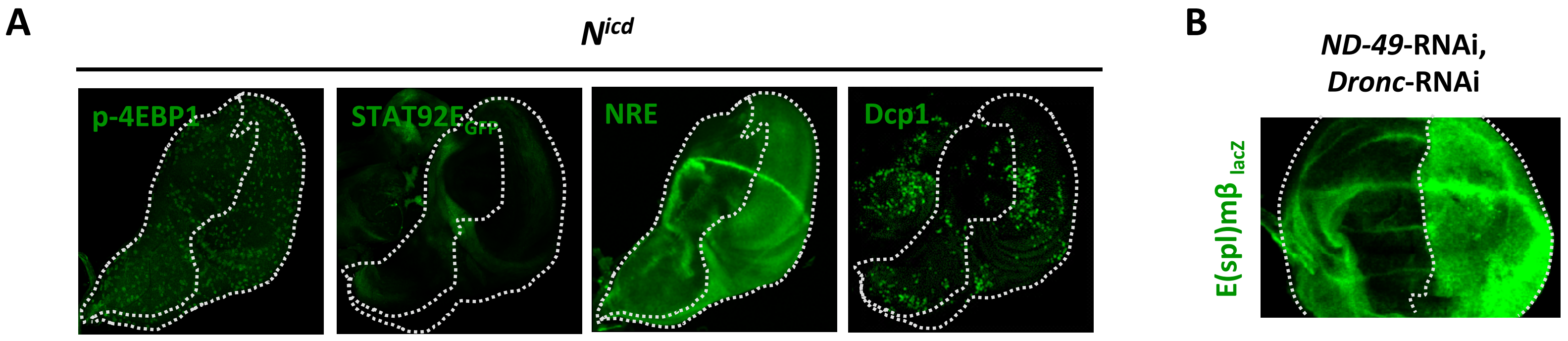

Supplementary figure S5: Complex I downregulation leads to Notch activation mediated by TOR.
(A) Activation of the Notch pathway through overexpression of Notch intracellular domain (N^{icd}) in the posterior compartment does not induce TOR (p-4EBP1) or JAK/STAT (STAT92E-GFP) pathways but it does induce apoptosis (Dcp1). NRE reporter served as positive control. (B) Blocking initiator caspase by *Dronc*-RNAi does not eliminate *E(spl)mb* expression observed in *ND-49*-RNAi.

A

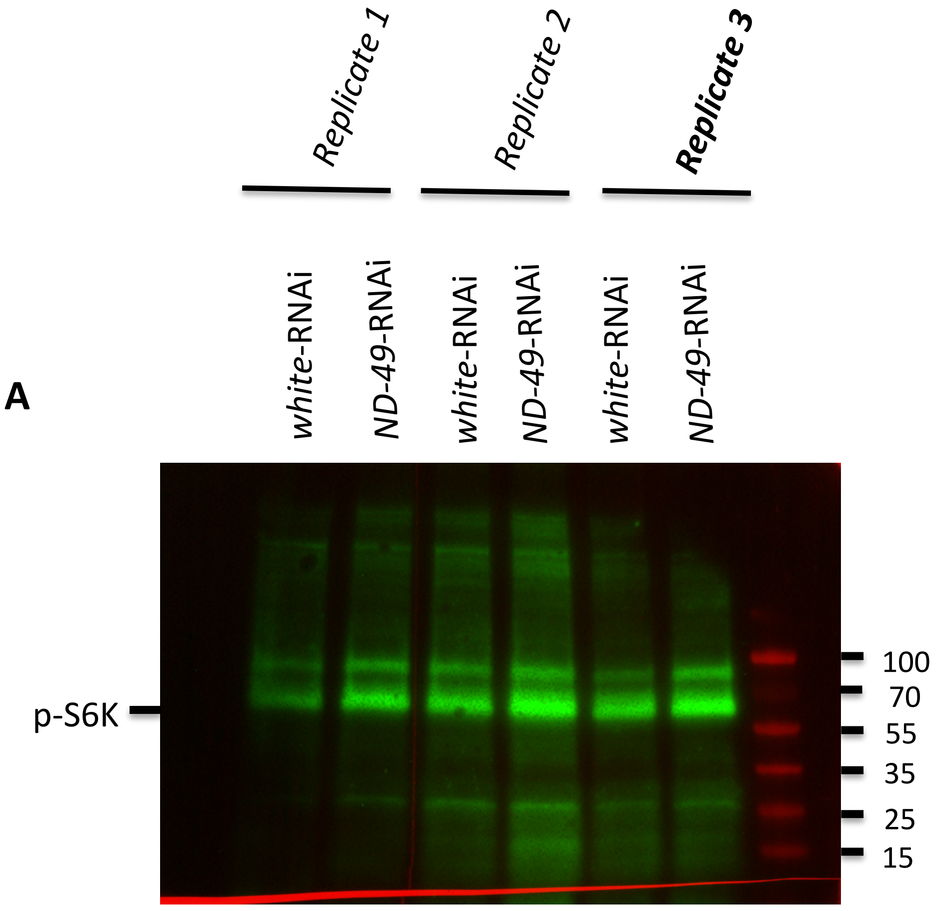

B

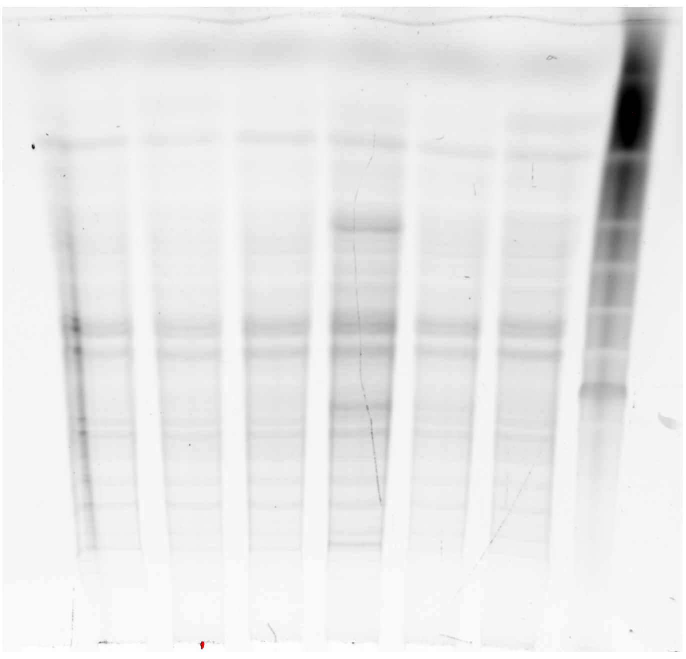

Supplementary figure 6: Uncropped original pictures of the western blot used for Fig. 1E and S1D. (A) The blot probed with phospho-Drosophila p70-S6K antibody (Cell Signalling 9209, 1:1000) and detected with the Clarity western ECL kit (Biorad) using HRP-conjugated secondary anti-rabbit antibody. Bottom part of the membrane was cut to be probed with a different antibody. (B) Total protein load assesed by fluorescent detection of proteins within the TGX Stain-Free gel (before transfer to membrane). Picture were taken and analyzed using the ChemiDoc imaging system (Biorad). Three biological replicates with the same experimental setup, replicate three was used for the figures.

Supplementary table ST1: Fly lines used in our study.

Fly strain	Description	Source
act-Gal4 *flip-out*	hsFLP; act>y+>Gal4, UAS-GFP to generate GFP labelled flip-out clones	BDSC26901, BDSC4411
UAS-Bsk	expresses Bsk (mammalian JNK orthologue)	BDSC 9310
UAS-BskDN	expresses a dominant negative form of Bsk	BDSC 6409
UAS-Bsk-RNAi	RNAi for Bsk (CG5680)	VDRC 34138 (GD)
UAS-Cyt-c-RNAi	RNAi for Cyt-c-p (CG17903), member of ETC complex IV	VDRC 106759 (KK)
UAS-DIAP1	expresses DIAP1, a negative regulator of initiator and effector caspases	BDSC 6657
UAS-Dronc-RNAi	RNAi for Dronc (CG8091), initiator caspase	VDRC 100424 (KK)
UAS-mit-GFP	GFP fused to a mitochondrial protein	BDSC 8442
UAS-Nicd (MH3)	Expresses Notch intracellular domain in the presence of Gal4	(Seugnet et al., 1997]
UAS-ND-49-RNAi	RNAi for complex I subunit ND-49	VDRC 108068 (KK)
UAS-ND-49-RNAi (2)	RNAi for complex I subunit ND-49	BL57499
UAS-ND-75-RNAi	RNAi for complex I subunit ND-75	VDRC 100733 (KK)
UAS-ND-75-RNAi (2)	RNAi for complex I subunit ND-75	BL27739
UAS-Notch-RNAi	RNAi for Notch receptor	BDSC 7078
UAS-p35	expresses the baculovirus p35 protein that blocks effector caspases	BDSC 5072
UAS-Tor-myc	expresses Tor	BDSC 53727
UAS-Tor-RNAi	RNAi for Tor	BDSC 34639
UAS-Tsc1/2	Expresses Tsc1 and Tsc2 in the presence of Gal4	(Tapon, et al, 2001)
UAS-Hif1-RNAi	RNAi for Hif1 (sima)	VDRC 106504
UAS-UQCR-RNAi	RNAi for UQCR (CG7580), member of ETC complex III	VDRC 101371 (KK)
CtBP-RNAi	RNAi for CtBP, part of Notch corepressor complex	VDRC 107313 (KK)
UAS-lacZ	control, drives lacZ in the presence of Gal4	BDSC8529
UAS-GFP-RNAi	control, drives GFP in the presence of Gal4	BDSC 9331
w1118	control	BDSC 5905
en-Gal4	drives UAS construct expression in the wing disc	
en-Gal4,Tub-Gal80ts	drives UAS construct expression in the wing disc at 29C	
hh-Gal4,Tub-Gal80ts	drives UAS construct expression in the wing disc at 29C	
aos-lacZ	aos-(p)-lacZ reporter of EGFR pathway activity	(Wessells et al., 1999)
E(spl)mβ-lacZ	p element containing HLHmbeta enhancer: P{E(spl)mβ-HLH-lacZ.0.9}	(Nellesen et al., 1999)
Hif1-RE-lacZ	Hif-1 responsive element driving lacZ expression	(Lavista-Llanos et al, 2002)
Ldh-GFP	GFP-based enhancer trap for Ldh	(Quiñones-Coello et al., 2007)
NRE-RFP	three copies of Gbe binding site + Su(H) binding sites of E(Spl)m8 gene	(Housden et al., 2012)
NRE-lacZ	three copies of Gbe binding site + Su(H) binding sites from E(Spl)m8 gene	(Furriols and Bray, 2001)
puc-lacZ	The pucE69 allele, P lacZ enhancer-trap insertion in the puc gene	(Ring and Martinez Arias, 1993)
STAT92E-GFP	10x STAT92E binding sites	(Back et al., 2007)
GstD1-GFP	ROS sensitive reporter	(Sykiotis et al., 2008)

BDSC - Bloomington Drosophila Stock Centre
VDRC - Vienna Drosophila Resource Centre

Supplementary table ST2: Antibodies used in our study.

Antibody	Source	Dilution
**Primary antibodies**		
rat-anti-Ci	DSHB, 2A1-s	1/100
mouse-anti-β-gal	DSHB, 40-1a-s	1/20
rabbit-anti-p-4EBP1	Cell Signaling Technology, 2855S	1/100
rabbit anti-Dcp1	Cell Signaling Technology, 9578S	1/100
rabbit anti-p-H3	abcam, ab5176	1/5000
rabbit anti-GFP	Thermo Fisher Scientific, G10362	1/10000
rabbit anti-dsRed	Clontech, 632496	1/500
rabbit anti-ATP5A	Abcam, 14748	1/500
**Secondary antibodies**		
Goat anti-mouse Alexa Fluor 488	Invitrogen, A11029	1/500
Donkey anti-mouse Alexa Fluor 555	Invitrogen, A31570	1/500
Donkey anti–mouse Alexa Fluor 647	Jackson, 715-605-150	1/500
Donkey anti–rabbit Alexa Fluor 555	Invitrogen, A31572	1/500
Donkey anti–rabbit Alexa Fluor 647	Invitrogen, A31573	1/500
Donkey anti-rat Cy3	Jackson, 712-166-150	1/500
Donkey anti-rat Cy5	Jackson, 712-175-153	1/500
